# Synergy of a Complimentary Ionic Biogel Network for Through-Hair Neurohaptics

**DOI:** 10.21203/rs.3.rs-5829714/v1

**Published:** 2025-02-06

**Authors:** Huanyu Cheng, Ankan Dutta, Abu Sayeed Biswas, Long Meng, Ethan Gerhard, Arantza Moreno Calva, Wanqing Zhang, Abu Musa Abdullah, Lana Joharji, Yuju Che, Jian Yang, Xiaogang Hu

**Affiliations:** The Pennsylvania State University; The Pennsylvania State University; The Pennsylvania State University; The Pennsylvania State University; The Pennsylvania State University; The Pennsylvania State University; The Pennsylvania State University; The Pennsylvania State University; The Pennsylvania State University; Shandong University; Westlake University; The Pennsylvania State University

## Abstract

Understanding the neural mechanisms underlying haptic sensations is crucial for advancing neuroprosthetics. However, achieving on-site amplification non-invasively through-hair neural recordings remains a significant challenge as it requires thermoreversible, bioadhesive, and semiconducting characteristics in the same material. Typical polymer composite compromises on complementary properties. To address this, we present a membraneless organelles - inspired ionic biogel that leverages liquid–liquid phase separation. This enables a unique synergy of complementary properties, including rapid thermoreversible transitions, p-type semiconductivity, thermoelectricity, enhanced electrochemical stability, self-healing, and bioadhesive capabilities. These characteristics enable to analyze the frequency dependence of event-related desynchronization during electrical stimulation over days mimicking the frequency response of mechanoreceptors sensation. This thermoresponsive, semiconducting ionic biogel also enables a phase-reversible, self-balancing, tip-shaped vertical organic electrochemical transistor with a high transconductance of 44 mS at 40°C. The ionic biogel demonstrates synergistic complementary properties to understand through-hair neurohaptics.

## Introduction

Transformative advancements in the field of neuroprosthetics offer hope to amputees and individuals with severe motor impairments by potentially restoring intuitive control and even sensation in prosthetic limbs^1^. Beyond motor control, transcutaneous electrical stimulation of peripheral nerves such as median and ulnar nerves has opened new possibilities to provide haptic feedback and enable amputees to experience projected touch sensations in their missing limbs^2,3^. However, current evaluations of these sensations often rely on subjective self-reports, limiting the precision and repeatability that are essential to further optimize prosthetic design^2^. Neurohaptics provides an objective measure of neural responses to sensory stimulation to enable refined, effective haptic feedback, which can seamlessly integrate neuroprosthetics with the user’s sensory perceptions^4,5^. The potential applications of neurohaptics also extend beyond prosthetics into the realm of augmented reality (AR) and virtual reality (VR), where sensory feedback could create immersive, interactive source-effector haptic experiences—presenting a highly promising commercializable approach^6^. This approach leverages projected sensations, enabling a seamless, comfortable AR/VR experience without compromising user immersion. Yet, as with neuroprosthetics, the field lacks consistent, objective data on neural responses to such haptic feedback, relying predominantly on subjective reports that limit the potential for standardization and optimization.

Understanding the neural mechanisms of haptic feedback during transcutaneous electrical nerve stimulation (TENS) holds promise to further advance the developments of neurohaptics. Event-related desynchronization (ERD) in the alpha or beta frequency band of electroencephalography (EEG) is a well-established biomarker of sensorimotor processing, frequently observed during tactile and mechanical stimuli such as vibration or pressure^7–9^. However, it remains unclear whether similar ERD responses are elicited through TENS, bypassing mechanoreceptors and directly activates sensory nerves. Identifying ERD in response to TENS would indicate that the sensorimotor cortex interprets these impulses as tactile inputs, providing an objective marker for tracking neural engagement and response fidelity in haptic interfaces. Moreover, long-term TENS invokes neuroplastic mechanisms that dynamically shape sensory perception over time^10,11^. Initially, repeated stimulation can intensify perceived sensations, potentially due to synaptic strengthening and recruitment of additional neural pathways^12^. However, with continued use, these sensations often diminish, reflecting neural habituation and circuit reorganization. This adaptation underscores the need for dynamic feedback systems that adjust stimulation to counteract habituation and sustain effective sensory feedback. Despite this potential, current approaches lack objective metrics for real-time monitoring of neural responses to haptic stimulation, and materials suitable for stable, long-term, and high-fidelity TENS and through-hair EEG recordings that could capture such responses.

**Figure 1:**
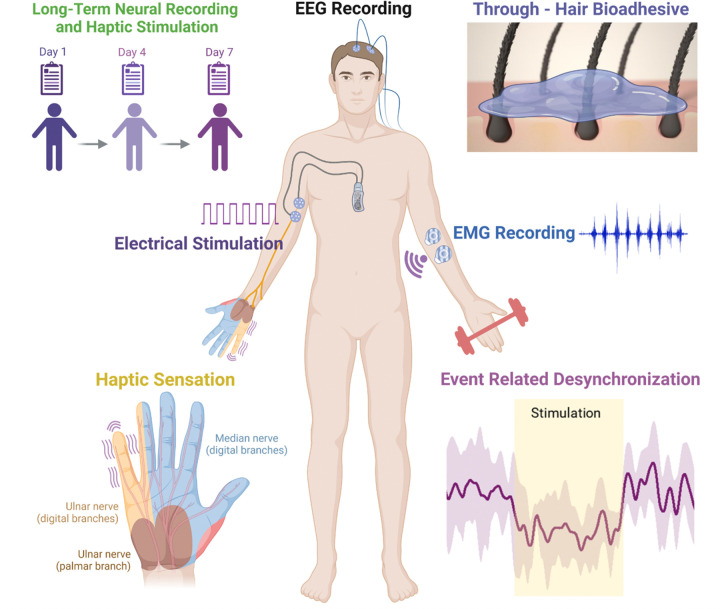
Schematic illustration of the experimental setup for neurohaptics using the ionic biogel with synergistic characteristics.

To address the persistent challenges in neurohaptics research of obtaining objective non-invasive EEG neural recording over the hair-covered motor cortex area (e.g. C3, C4, Cz) during haptic sensation, we introduce an ionic biogel network system with thermoresponsive and semiconducting characteristics, capable of concurrent high-fidelity, long-term through-hair neural recording and TENS (Fig. 1a). The ionic biogel synergistically combines two material systems with independent yet complementary bonding mechanisms to result in a softer material with faster sol-gel transitions and enhanced mechanical, electrical, and electrochemical properties **(Extended** Fig. 1a). In particular, the electrostatic interactions between the poly(3,4-ethylenedioxythiophene) poly(styrene sulfonate) (PEDOT:PSS) and a plasticizer such as ionic liquid, here 1-ethyl-3-methylimidazolium bis(trifluoromethylsulfonyl)imide (EMIM-TFSI) (ionic system)^13^ are combined with the hydrogen-bonding network of the gelatin-glycerol-salt hydrogel matrix (biogel system)^14^. The resulting ionic biogel addresses the challenges associated with through-hair EEG and TENS in three key aspects. First, its thermoresponsive properties (from the biogel system) provide the ionic biogel with the rapid gel-sol transition to conform to the scalp/skin upon gentle heating, thus maintaining stable, robust contact for reliable neural recording and haptic stimulation. Second, the viscoelastic flow behaviour (from the biogel system) of the ionic biogel maximizes the contact area to reduce the skin contact impedance and improve signal-to-noise ratios even upon motions due to the bioadhesive properties. Although the thermoresponsive behavior of a biogel-based system has been previously demonstrated for through-hair EEG recording, prior material systems do not possess the rapid thermoreversibility, high water retention, and electrochemical stability necessary for sustained operation over days to weeks^14,15^. Third, leveraging the semiconducting properties of PEDOT:PSS (from ionic system)^16–19^, the ionic biogel can function as a thermoreversible p-type semiconductor to enable the integration of the organic electrochemical transistors (OECTs) as through-hair active electrodes with on-site amplification. Previously, OECTs have been employed for high-fidelity neural recordings^20,21^, but they lack thermoreversible and bioadhesive characteristics to enable a robust, through-hair EEG recording. Collectively, the unified properties of this ionic biogel enable the synthesis of bioadhesive semiconducting hydrogel-based interfaces exhibiting rapid gel-sol transitions—providing stable, high-quality, long-term through-hair EEG recordings and haptic stimulation.

Typical polymer blending often yields a mere averaged combination of favourable properties, rather than achieving true functional synergy. In contrast, harnessing the synergy inherent in complementary material systems requires capitalizing on the spatial and compositional segregation characteristic of liquid–liquid phase separation (LLPS)^22–25^. Membraneless organelles (MLOs) in biological cells exemplify this principle: by partitioning biomolecules into distinct coexisting phases, MLOs leverage metastability and rapid, reversible transitions to integrate and optimize diverse functionalities^26,27^. Drawing inspiration from nature’s optimized LLPS-driven functionality of MLOs, the design of phase-separated ionic biogels strategically fuses dissimilar but complementary phases to realize synergy. One phase, formed from the PEDOT:PSS/EMIM-TFSI system, imparts high ionic conductivity, p-type semiconducting behavior, thermoelectricity, robust water-retention characteristics, and high electrochemical stability, enabling long-term, high-fidelity neural recording, and precise electrical stimulation. The other phase, the biopolymeric gelatin–glycerol–salt matrix, introduces biocompatibility, bioadhesive properties, self-healing capabilities, and thermoresponsive behaviour, and also contributes to the thermopower. Together, these coexisting phases maintain a phase-separated metastable state that allows for rapid, thermoreversible sol-gel transitions, facilitating conformality. This synergy yields an MLO-inspired, phase-separated ionic biogel with a unified combination of properties—metastability, thermoreversibility, porosity, p-type semiconductivity, high air permeability, long-term stability, injectability, and strain insensitivity.

### Characterisation of the synergistic ionic biogel network

The complementary properties of the ionic biogel stem from the synergistic interplay of phase-separated PEDOT:PSS/EMIM-TFSI ionic system and the gelatin-glycerol-salt hydrogel matrix **(Extended** Fig. 1b). The independence of these two material systems **(Supplementary Note 1)** minimizes unfavourable inter-network interactions, as their primary bonding mechanisms—electrostatic interactions (in ionic system) and hydrogen bonds (in biogel system)—operate within distinct individual phases. This arrangement exhibits metastability and phase separation, typically observed in MLOs, where compartmentalization arises from weak, dynamic interactions^24,26^ **(Supplementary Note 2)**. Initially, the mixture forms an amorphous state due to kinetic constraints and limited molecular mobility at room temperature; upon heating, increased chain mobility reduces the Gibbs free energy of mixing (${\Delta \;G}_{mix}$), leading to partial miscibility^28^. Upon cooling, the spatial separation **(Figure S1)** and difference in energy scales elevate the Gibbs free energy of mixing ${\Delta \;G}_{mix}$, favouring phase separation and the formation of a metastable MLO-inspired composite material **(Supplementary Note 3)**. The stable open electrochemical potential of −40 mV for > 7 hours **(Figure S2)** indicates that the interface does not spontaneously homogenize because that would require overcoming significant energy barriers: interfacial tension, rearrangement of polymer chains, re-solvation or displacement of ions, and possibly changes in polymer redox states^29^ **(Supplementary Note 4)**. The molecular interactions between the gelatin-glycerol-salt hydrogel matrix and the PEDOT:PSS/EMIM-TFSI ionic system can be elucidated from a comprehensive series of characterizations **(Supplementary Note 5, Figures S3-S8, Extended** Figs. 2a-2f).

## Results and Discussion

**Figure 2:**
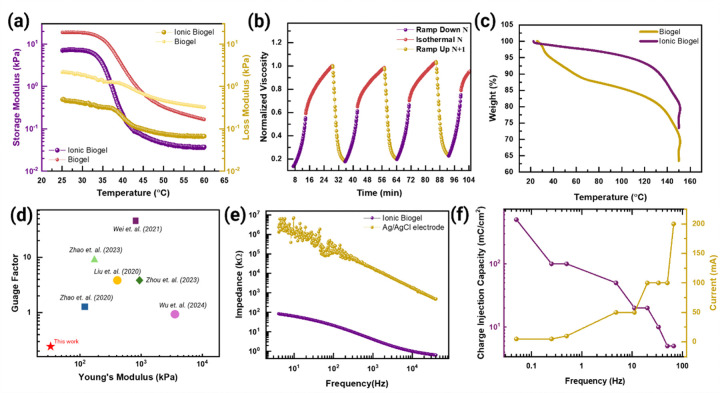
Characterization of Ionic Biogel showing robust sol-gel transition. **(a)** Comparison in storage and loss moduli as a function of temperature between non-ionic and ionic biogel. **(b)** Variation in the normalized viscosity from a ~1-day ionic biogel during repeated temperature cycles of ramp-up, ramp-down, and isothermal conditions. **(c)** Comparison in the thermogravimetric analysis (TGA) between the biogel and ionic biogel. **(d)** Comparison of strain-insensitivity and Young’s modulus among reported hydrogels^31–36^. None of them reported self-healing properties, except for this work. **(e)** Comparison in the skin contact impedance between the ionic biogel and Ag/AgCl on beard. **(f)** CIC and permissible current amplitude across the frequency range from 0.05 Hz to 67 Hz.

**Figure 3:**
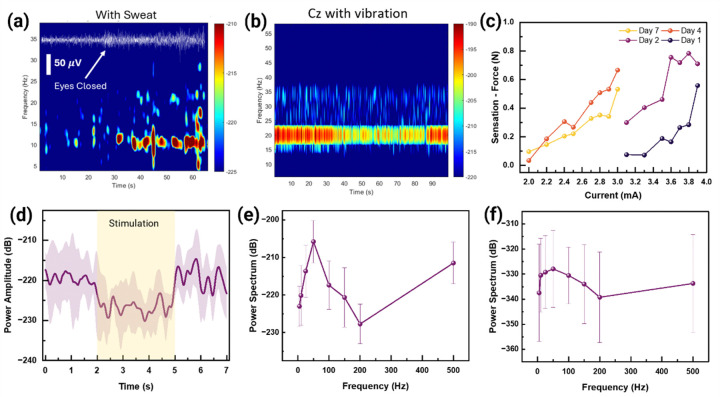
EEG characterization during mechanical and electrical stimulation. **(a)** Power spectrogram showing increased power in the alpha regime during the eye-closing state. Schematic of the wearable electrical stimulator array placed on the arm. **(b)** Power spectrogram showing ERD during sensory mechanical vibration at Cz locations. **(c)** Variation of (force) sensation from ulnar nerve stimulation across 7 days. **(d)** Power spectrogram of EEG during electrical stimulation at 150 Hz. Power spectrum of EEG during haptic sensation applied at different frequency (5 Hz – 500 Hz) on **(e)** Day 1 and **(f)** Day 7.

One of the essential characteristics of the ionic biogel is a sharper gel-sol transition at ~ 40°C to ensure rapid extrusion of the material onto the scalp. Metastability, akin to the behaviour observed in MLOs, can be experimentally validated through rapid phase transitions captured in rheological measurements^26^. The storage modulus of the ionic biogel composite transitions from 7 kPa at room temperature to 3.4 kPa at scalp temperature (35°C) and then to a very low modulus of 60 Pa at 45°C (Fig. 2a). This transition facilitates mechanical adaptability to flow and exert minimal strain on the scalp and surrounding hair, compared to normal biogel^14,15^ **(Supplementary Note 6, Figures S9-S10, Extended** Figs. 3a-3b), following a the modified-Ellis equation for the temperature-sweep rheology **(Supplementary Note 7)**. A cyclic thermal ramping protocol is employed to investigate the material’s ability to recover its initial rheological characteristics (Fig. 2b), demonstrating its metastable characteristic **(Supplementary Note 8, Figures S11-S16, Exten**ded Fig. 3c), which can be modelled as a spring-damper model with different growth time constants to account for decay and reversibility **(Supplementary Note 9)**.

The ionic biogel has distinct mechanical and adhesive properties in shear and normal direction **(Supplementary Note 10, Figures S17-S19, Extended** Fig. 3d). The ionic biogel shows high water retention property (~ 97% at 70°C) (Fig. 2c) and water vapour transmission rate (~ 1417 g/m^2^/day) **(Supplementary Note 11, Figure S20, Extended** Fig. 3e). The synergistic interaction of thermodiffusion and thermogalvanic effects^30^ in ionic biogel composite drives a high Seeback coefficient of 21 mV/K (till 3 K change) and then 4 mV/K (till 8 K change), offering promising application opportunities in low-grade thermal energy harvesting and wearable thermoelectric devices **(Supplementary Note 12, Extended** Fig. 3f). The porous nature of the ionic biogel from phase separation along with the electrostatic interaction of PEDOT:PSS/ionic liquid makes it much softer than the normal biogel (**Supplementary Note 13, Figures S21, S22)**. One of the most important benefits of phase-separation is the strain-insensitivity of the ionic biogel which only increases its resistance by 100% after reaching its strain limit of 380%, which represents the record high strain insensitivity among all types of hydrogels to our knowledge^31–36^ (Figs. 2d, S23). The material shows noticeable changes (> 1%) in resistance only after > 30% strain. The material also shows negligible hysteresis with resistance returning to its original value with only a 1% change.

The performance of the through-hair electrophysiology hinges on the skin contact impedance through the hair **(Supplementary Note 14, Figures S24-S29, Extended** Figs. 4a-4d), where the impedance of 1.5 kΩ (diameter ~ 2 cm) from the ionic biogel at 4 kHz is more than three orders of magnitude smaller than that of 4.4 MΩ from the commercial Ag/AgCl electrode (Fig. 2e). The low skin-contact impedance shows a robust EMG signal during flexing **(Supplementary Note 15, Figures S30-S34)**. Charge injection capacity (CIC) is one of the most crucial parameters for evaluating the performance of electrical stimulation in neuroprosthetics^31,37,38^. The CIC of the ionic biogel is 10 mC/cm^2^ at 33 Hz and 5 mC/cm^2^ at 67 Hz (Fig. 2f), which are comparable to existing materials used for similar applications^31,38^ **(Supplementary Note 16, Figures S35-S40, Extended** Figs. 4e-4f).

The thermoresponsive and conducting properties of the ionic biogel ensure stable, high-quality, long-term signal acquisition and reliable stimulation, even with different hair types or skin oils. An increase in EEG power amplitude during closed eyes state is observed with (Fig. 3a) and without sweat **(Figure S41)** compared to open eyes state. The efficacy of the ionic biogel electrode is demonstrated through ERD in the recording of neural responses associated with motor cortex activity during mechanical or natural sensation **(Supplementary Note 17, Figures S42-S46, Extended Figs. 5a-5e)**. The EEG power spectrum at C3 and Cz during sensory mechanical vibration (using a vortex vibrator) reveals a similar ERD trend: a reduction in beta power amplitude at Cz during weight repetitions, but no ERD reflected at C3 ^9^ (Fig. 3b, **Extended Fig. 5f)**.

ERD has been known to occur during hand or finger movement^7,8^, which is also consistent with mechanical vibration. However, to the best of our knowledge, no prior studies have correlated ERD with haptic sensations induced by TENS of the ulnar nerve. In this context, neural responses during cutaneous electrical stimulation are evaluated with a wearable stimulator array **(Extended Fig. 6)** consisting of an 8×2 grid of electrodes arranged with a constant pitch of 1.5 cm to cover the right arm **(Supplementary Note 18, Figure S47)**. On Day 1, the perceived force sensation is increased from 0.07 N to 0.5 N as the stimulation current applied to the ulnar nerve is increased from 3.1 mA to 3.9 mA, till the subject reports perceived pain (pain threshold) (Fig. 3c). Notably, on Day 2, the baseline force perception at the same stimulation current of 3.1 mA increases to 0.3 N, suggesting a possible sensitization or adaptation effect within the nervous system. A decrease in the pain threshold (current amplitude at which the pain onsets) is observed on Days 4 and 7, with similar force sensations of ~ 0.1 N being elicited at a significantly lower stimulation current of 2 mA. Specifically, on Day 4, a stimulation current of 3 mA can induce a sensation force that is about 842% of the value induced by the 3.1 mA stimulation on Day 1. Although the sensation force on Day 4 exhibits a similar trend and range to that observed on Day 7, there is a slight increase of ~ 20% at 3 mA (the higher end of the current range). These findings indicate potential neuroplastic changes occurring over repeated stimulation sessions, possibly involving peripheral sensitization mechanisms that enhance neural responsiveness to electrical stimuli^10,11^.

A significant challenge in EEG recordings during simultaneous electrical stimulation is the introduced artifacts, particularly charging artifacts resulting from current flow through the body, which can produce high-amplitude voltages (tens of millivolts range) at the scalp and contaminate the EEG signal (in microvolts range). By positioning the ground electrode along the cervical region and ulnar nerve pathway rather than behind the ear, these artifacts are reduced from approximately 40 mV to 4 mV. The artifacts are further reduced by sequential post-processing methods such as empirical mode decomposition (EMD)^39^, bandpass/notch filters, and downsampling **(Supplementary Note 19)**. The artifact removal is generally performed using Independent Component Analysis (ICA)^40^ that leverages the statistical independence of multiple channels. However, as the number of electrodes is two in this work, ICA’s performance is restricted. Therefore, EMD is utilized to decompose the signal into several intrinsic mode functions (IMFs) with the first mode corresponding to the stimulation artifacts. Similar to mechanical stimulation, ERD is also observed during electrical stimulation, which is marked by a reduction of power in alpha-wave, averaged across trials for all amplitudes (Fig. 3d). Different frequency (5–500 Hz) of electrical stimulation (of amplitude of 3.7 mA) is elicited to record the change of alpha power in EEG during stimulation (Fig. 3e, **Supplementary Note 20)**. The neural response (alpha power of EEG) across different frequencies increases, reaching a maximum at 50 Hz and also around 500 Hz, corresponding to the resonant frequencies of mechanoreceptors such as Meissner Corpuscles and Pacinian corpuscles^2,41,42^ respectively, as shown in other methods during mechanical/natural stimulation **(Supplementary Note 21)**. This power attenuation **(Figure S48)** and its spectrum across different frequencies persist on Day 7 (Fig. 3f).

**Figure 4:**
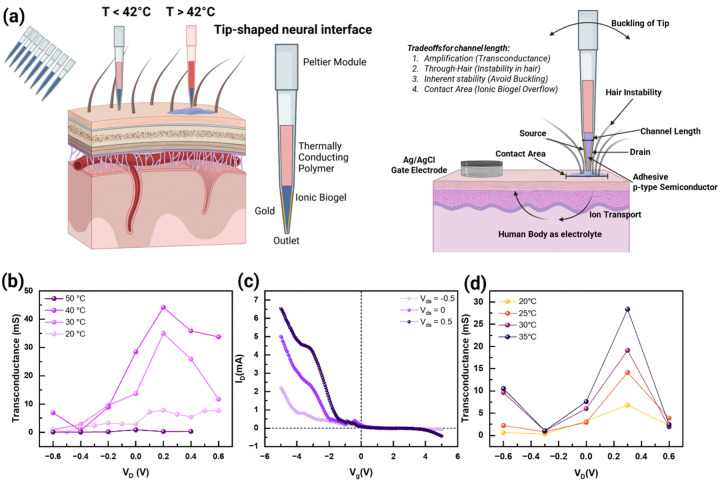
Semi-conducting nature of Ionic Biogel. **(a)** Schematic of tip—shaped neural interface for phase-transition, organic electrochemical transistor (OECT). **(b)** Transconductance of tip-shaped OECT at different temperatures in ionic liquid as an electrolyte, crossing the phase-transition temperature. **(c)** *I*_*d*_*-V*_*g*_ transfer characteristics of OECT in Phosphate Buffer Solution (PBS) solution. **(d)** Comparison in the transconductance of the temperature-dependent phase-reversible tip-shaped OECT in PBS as an electrolyte, without crossing the phase-transition OECT: 20, 25, 30, and 35°C.

Designing through-hair active electrodes with low skin contact impedance necessitates navigating several interdependent constraints, especially when utilizing thermoresponsive, semiconducting materials that undergo gel-sol phase transitions. The multiphysics nature of the problem arises from the interplay between thermal dynamics, phase transitions, fluid mechanics, solid mechanics, and transistor physics. Notably, the viscosity of the ionic biogel depends on temperature, influencing fluid flow and ion transport within the ionic biogel domain. To simplify without the loss of generality, we focus on optimizing a tip-shaped neural interface, where the tip diameter corresponds to the channel length *L* between the source and drain of a vertical OECT (Fig. 4a). In the vertical OECT localized at the tip with gold source/drain electrodes, the column is filled with a thermally conductive graphite-PDMS composite to enhance thermal transport, actively regulated by a Peltier module atop the assembly. The channel length or the diameter of the tip can be optimized as around 2.8 mm, for a 1 cm long tip which self-balances itself **(Supplementary Note 22, Figure S49)**, also confirmed from COMSOL Multiphysics simulation **(Supplementary Note 23, Extended Figs. 7a-7c)**. The electrical impedance and capacitance of the tip-shaped OECT are measured to be approximately 120 Ω and 40 μF at 100 Hz and room temperature, respectively **(Supplementary Note 24, Figure S50)**. The decrease in the skin contact impedance improves the signal coupling **(Supplementary Note 25)**.

The transconductance of the OECT exhibits a strong dependence on temperature and the choice of electrolyte. Phosphate-buffered saline (PBS), a conventional electrolyte for OECTs, tends to dehydrate at elevated temperatures, impairing ionic conductivity and device performance. To address this limitation, the ionic liquid EMIM-TFSI is employed as the electrolyte due to its high thermal stability and excellent ionic conductivity. At a drain voltage of 0.2 V, the OECT displays a maximum transconductance of 7.8 mS at 20°C. This transconductance increases to 35 mS at 30°C and further to 44 mS at 40°C (Fig. 4b). The enhancement in transconductance with temperature can be attributed to several factors, including increased ionic mobility, thermoresponsive gelatin-glycerol matrix (decreased viscosity at higher temperatures to allow for more rapid ion diffusion and improved coupling between the gate and channel), and enhanced electronic conductivity (from increased chain mobility and enhanced π–π stacking interactions). However, upon surpassing the phase transition temperature of the gelatin-glycerol matrix at approximately 42°C, the transconductance is dramatically decreased to 0.3 mS at 50°C. This sharp decline is attributed to the loss of structural integrity of the hydrogel matrix as it transitions from a gel to a sol state, causing the ionic biogel to flow. The phase transition can be avoided by operating below the phase-transition temperature, enabling phase-reversible OECTs **(Figure S51)**. Notably, at a negative drain voltage of − 0.4 V, the transconductance remains low (< 3 mS) across the entire temperature range. This asymmetry in device behaviour suggests that the OECT is more efficient in modulating channel current through hole depletion rather than accumulation, consistent with the p-type semiconducting nature of PEDOT:PSS. A similar trend is observed when PBS is used as the electrolyte (Figs. 4c, 4d), although the overall performance is inferior compared to EMIM-TFSI. The dehydration of PBS at higher temperatures reduces ionic conductivity and impairs the gating efficiency, leading to lower transconductance values. This comparison highlights the critical role of the ionic liquid EMIM-TFSI in maintaining high ionic conductivity and thermal stability, essential for the OECT’s operation at elevated temperatures.

## Conclusion

Looking ahead, this work sets a foundation for further exploration into complementary material systems to develop next-generation neurointerfaces with enhanced functionality. By leveraging synergistic interactions, such as those provided by the combination of the hydrogen-bonded gelatin matrix with the PEDOT:PSS/ionic liquid domains, designs can further tune properties such as ionic conductivity, charge retention, mechanical adaptability, and durability to specific neurophysiological needs. The introduction of phase-separated, porous architectures within these materials could further augment their electrochemical performance, enabling seamless transitions between ionic and electronic conductivity within a three-dimensional, volumetric framework. Such advancements are expected to drive significant progress in the emerging field of neurohaptics, where finely-tuned materials can bridge the gap between sensory feedback and neural stimulation, offering users immersive and responsive experiences. By creating adaptive, responsive materials that closely mimic biological tissues, we are better positioned to study sensory processing, adaptation, and long-term neural plasticity. This work underscores the transformative potential of bioinspired, multifunctional materials in enabling new frontiers in neural engineering, paving the way for neuroprosthetics and wearable neurotechnologies that seamlessly integrate with human physiology.

## Methods

### XPS

XPS experiments were performed using a Physical Electronics VersaProbe III instrument equipped with a monochromatic Al kα x-ray source (hν = 1,486.6 eV) and a concentric hemispherical analyzer. Charge neutralization was performed using both low-energy electrons (< 5 eV) and argon ions. The binding energy axis was calibrated using sputter cleaned Cu (Cu 2p3/2 = 932.62 eV, Cu 3p3/2 = 75.1 eV) and Au foils (Au 4f7/2 = 83.96 eV).^43^ Peaks were charge referenced to the CHx band in the carbon 1s spectrum at 284.8 eV. Measurements were made at a takeoff angle of 45° with respect to the sample surface plane. This resulted in a typical sampling depth of 3–6 nm (95% of the signal originated from this depth or shallower). Quantification was done using instrumental relative sensitivity factors (RSFs) that account for the x-ray cross-section and inelastic mean free path of the electrons. On homogeneous samples major elements (> 5 atom%) tend to have standard deviations of < 3% while minor elements can be significantly higher. The analysis size was ~ 200 μm in diameter.

### Raman Spectroscopy

Raman spectroscopy was employed to investigate the surface composition and structural properties of the composite using a Horiba LabRAM HR Evolution confocal microscope equipped with a 785 nm laser and a 300 g/mm grating. The characterization was conducted across various temperatures to assess temperature-dependent changes.

### FTIR

FTIR measurements were conducted using a Bruker V70 spectrometer (wavenumber range: 4000 to 600 cm⁻¹, resolution: 6 s⁻¹) to examine the chemical bonding and identify organic and inorganic components in the modified nanocomposite films through infrared light. The characterization was performed at different temperatures to evaluate temperature-dependent changes.

### Biocompatibility Test

Hydrogel samples were cut into circular films with 10 mm diameter and added to PBS to prepare hydrogel extracts, and 1.0 mL of the extract was put into a 24-well plate for UV irradiation. For each well containing hydrogel extract, 2×10^4^ MCF-10A was added and the proliferation medium was supplemented to 1.0 mL. For the wells without material, 2×10^4^ MCF-10A was also added and the proliferation medium was supplemented to 1.0 mL as the control group. The 24-well plate containing MCF-10A and the extract was incubated in a cell culture incubator, and 200 μL of proliferation medium was added to each well every two days. The proliferation of MCF-10A on the hydrogel extract on days 1 and 2 after co-culture was determined using MTT. After adding the FDA, the staining results were observed using a fluorescence microscope.

### Mechanical Testing

#### General Procedures

All mechanical and adhesive testing was conducted on an Instron J5966 testing frame (Instron, Binghamton, NY, USA) with a 1 kN load cell and utilizing Bluehill Universal mechanical testing software (Instron).

#### Compression Testing

Compression testing between parallel plates (Instron) was carried out on hydrogel specimens with a diameter of 8 mm and thickness of 1 mm to 90% strain at a strain rate of 1.3 mm/min. Initial modulus and peak stress at 90% strain were calculated from the stress profile over the initial 10% strain and the maximum stress value, respectively.

##### Tensile Testing:

Tensile testing was carried out using pneumatic grips (Instron) on hydrogel samples (length × width × thickness: 3 cm × 1 cm × 1mm) at a strain rate of 50 mm/min. Initial tensile modulus, peak stress, and peak strain were calculated from the stress profile over the initial 10% of strain and the maximum stress and strain values, respectively.

#### Adhesive Testing

Lap shear adhesive testing was carried out using pneumatic tensile grips. Strips of porcine tissue (1 cm width) were adhered in a lap configuration with an adhesive area of 1 cm^2^ and placed within the tensile grips (with the adhered region outside of the gripped area). The tensile force was then applied at a rate of 5 mm/min until adhesive failure and the maximum adhesive force was recorded. 90° adhesive peel testing was carried out by first gluing a porcine tissue strip (1 cm width) to a flat acrylic plate, which was then attached to the bottom compression plate of the mechanical testing frame using vice grips. Another strip of porcine tissue was adhered to this first piece using the appropriate hydrogel adhesive to create an adhesive area of 1 cm^2^. The other, free end of the second strip was then gripped by the top pneumatic grip of the testing frame, after which a tensile force was generated to the top to achieve the 90° peel, with adhesive force measured as above.

### Rheological Testing

#### General Procedures

All rheological testing was conducted on a DHR-2 rheometer (TA Instruments, New Castle, DE, USA) using TRIOS software (TA Instruments) in a 20 mm parallel plate configuration (sandblasted plate faces) with a Peltier plate to control temperature. Hydrogel samples with a diameter of 20 mm and height of 1 mm were used at 25°C unless stated otherwise.

#### Shear Sweep

Shear sweeps were first conducted to establish the linear response region from 0.01 to 1,000% strain at a frequency of 1 Hz.

#### Frequency Sweep

Frequency sweeps were conducted from 0.1 to 100 Hz within the linear response region (0.05% strain).

#### Temperature Sweep

Temperature sweeps were conducted from 25 to 60°C with a ramp rate of 1°C/min (1 Hz and 0.05% strain).

##### Cyclical Recovery Test:

Cyclic recovery tests were conducted in 3 phases: an initial ramp from 25 to 60°C at a rate of 5°C/min (1 Hz and 0.05% strain), a cooling ramp from 60 to 25°C at a rate of 5°C/min (1 Hz and 0.05% strain), and a recovery interval at 25°C for 900 s (1 Hz and 0.05% strain). The above 3 phases were repeated over successive 4 cycles.

### Stimulation Procedure

A 26-year-old male human subject participated in the stimulation experiment. During the experiment, the subject was comfortably seated in a chair. The electrical stimuli were delivered via a set of 2×8 gel-based electrodes (Fig. 6 (b)). Each electrode with a diameter of 1 cm was replaced with the reported ionic biogel to enhance conductivity. The electrodes were positioned along the medial portion of the right upper arm, just beneath the short head of the biceps brachii, targeting the area near the median and ulnar nerves. This placement aligned with the way extending from the center of the armpit to the inner side of the elbow (medial epicondyle of the humerus), ensuring placement over the nerve pathways located below the skin. To deliver the electrical stimuli via a specific pair of electrodes (Fig. 6(b)), a custom-built MATLAB interface to control a switch matrix (Agilent Technologies, Santa Clara, CA) for electrode pair selection was used. The MATLAB interface can also configure the stimulation parameters of the stimulator (STG4008, Multichannel Systems, Reutlingen, Germany). Specifically, single biphasic rectangular pulses (Fig. 2(a)) were delivered to selected electrodes with a pulse width of 200 μs and a stimulation frequency of 150 Hz. To indicate the force evoked by stimulation, the subject’s right hand was placed on a force load cell (LCM201–100N, Newark Electronics, Chicago, IL) with a sampling frequency of 1,000 Hz, allowing the subject to apply varying levels of pressure with the index finger in response to force evoked by stimulation.^44^

A grid search was initially performed to identify a pair of electrodes that could effectively induce flexion in at least one finger, while minimizing wrist movement and avoiding any discomfort. Once identified, this electrode pair was used for subsequent experiments. Multiple 3-s stimulation trials were conducted, with the pulse amplitude gradually increased until the subject reported discomfort. The measured forces during the plateau period were averaged to indicate the force evoked by stimulation. The evaluation of the long-term usability of the reported ionic biogel involved a series of experiments over the span of one week, specifically on Days 1, 2, 4, and 7.

### Materials

Gelatin from porcine skin powder with gel strength 300 g Bloom Type A, Glycerol 99%, sodium chloride (NaCl), and the ionic liquid 1-Ethyl-3-methylimidazolium bis(trifluoromethylsulfonyl)imide with a concentration of 98% (HPLC) were purchased from Sigma Aldrich. PEDOT:PSS (PH 1000) in aqueous solution was purchased from Ossila.^13–15^ For PDMS, Sylgard 184 Elastomer Base and its Curing Agent from Electron Microscopy Sciences were used at a 10:1 ratio.

### Preparation of Biogel

#### Original Biogel

The biogel was prepared by dissolving 0.11 grams of sodium chloride in 1 mL of water. Afterward, 0.75 grams of glycerol were added and mixed, followed by adding and thorough mixing of 0.5 grams of gelatin.^14,15^ The solution was left for 2 hours so the gelatin could absorb all the components and then heated in an oven at 80°C for 45 minutes. At this time, the biogel could be used as a gel with relatively liquid and highly adhesive properties. For storage or later use, the gel was left at room temperature for around 6 hours to solidify. The biogel could be heated at any time afterward to reacquire its liquid form.

#### Ionic Biogel

The ionic biogel was prepared by dissolving 0.11 grams of sodium chloride in 1 mL of PEDOT:PSS in an aqueous solution. Afterward, 1 mL of ionic liquid was added and mixed thoroughly.^13^ After 5 mins, 0.75 grams of glycerol were added and mixed, followed by adding and thorough mixing of 0.5 grams gelatin. The solution was left for 2 hours so the gelatin could absorb all the components and then heated in an oven at 80°C for 45 minutes. At this time, the ionic biogel could be used as a gel in a relatively liquid form. For storage or later use, the resulting ionic biogel was left at room temperature for around 6 hours to solidify. The ionic biogel could be heated at any time afterward to reacquire its liquid and adhesive properties.
